# Editorial: SARS-CoV-2 vaccines beyond the pandemic era, volume II

**DOI:** 10.3389/fmed.2026.1939146

**Published:** 2026-08-03

**Authors:** Shisan Bao, Farid Rahimi, Ritthideach Yorsaeng

**Affiliations:** 1 Centre for Evidence Based Medicine, Gansu University of Chinese Medicine, Lanzhou, China; 2 Research School of Biology, The Australian National University, Acton, ACT, Australia; 3 Center of Excellence in Clinical Virology, Department of Pediatrics, Faculty of Medicine, Chulalongkorn University, Bangkok, Thailand

**Keywords:** immunity, pendamic, public health, SARS-C0V-2, vaccination

Although WHO declared an end to the COVID-19 Public Health Emergency of International Concern in May 2023, SARS-CoV-2 continues to circulate globally and evolve concurrently with waning host or population immunity and changing susceptibilities of hosts. Vaccination therefore is crucial for disease prevention, particularly in populations prone to severe illness or severe complications. Thus, collective research has shifted focus from rapid development and deployment of vaccines to optimizing vaccination strategies, understanding long-term immunity, evaluating vaccine safety, and establishing health measures to protect the public health.

*SARS-CoV-2 Vaccines Beyond the Pandemic Era – Volume II* was assembled to capture these emerging directions and collects articles covering immune protection, vaccine effectiveness and safety, maternal and neonatal immunity, high-risk populations, and evaluation of real-world epidemiological evidence. While each study tackles a different aspect of COVID-19 vaccination, together they highlight a common objective: generating evidence that informs vaccination policy for managing the circulating SARS-CoV-2 beyond its pandemic phase.

Instead of cataloging individual studies, we summarize the broader themes emerging among the collected articles: advancing our understanding of protective immunity, developing new vaccination strategies, importance of population-specific vaccination, ongoing safety surveillance of vaccines, hosts' pre-existing immune factors influencing disease outcomes, mechanisms of COVID-19 immunopathology, and methodological challenges associated with observational studies that evaluate long-term outcomes.

The Research Topic captures also the evolution and refinement of vaccine design. Heterologous booster regimens, “topical” respiratory mucosal vaccines, and updated variant-specific vaccines are explored to broaden immune protection against the SARS-CoV-2 variants. Among several contributions, hybrid immunity—generated through combination of vaccination and natural infection—emerges as an important concept for understanding durable and broad immune protection against evolving SARS-CoV-2 variants. Instead of superseding, these approaches intend to complement existing vaccination strategies by strengthening immunity because first-generation vaccines were limited, particularly in preventing infection at the respiratory mucosa (supporting the innate immune systems).

Several articles focus on populations that are vulnerable to COVID-19. For example, pregnant individuals and newborns in studies examining vaccine immunogenicity, maternal antibody responses, transplacental antibody transfer, and maternal and neonatal health outcomes. Such evidence highlights the vaccination benefits during pregnancy while emphasizing that completing the recommended vaccine schedules is important for maximizing protection of both gravid mothers and newborns. The accompanying discussion of vaccine hesitancy highlights that generating high-quality evidence is only one aspect of successful vaccination programs; effective communication and public education, and equitable access are equally important.

Host factors that influence immune responses also receive considerable attention. Reportedly, for example, patients with resolved hepatitis B infections experienced enhanced inflammatory responses and poorer clinical outcomes following COVID-19 affliction. Although these findings require confirmation in large and diverse cohorts, they reinforce that pre-existing immune status may contribute to the marked heterogeneity observed with COVID-19 and should be considered when evaluating disease risk and responses to vaccines.

As vaccination programs mature, long-term monitoring of safety has become increasingly important. This Research Topic includes studies of adverse events following immunization and exploratory studies examining mortality patterns after repeated booster vaccination. Such studies provide valuable real-world evidence but also illustrate the limitations of observational studies. Particularly, age differences, comorbidities, healthcare access, socioeconomic factors, and other potential factors can substantially influence observed associations. Several authors appropriately emphasize that their findings must not be interpreted as evidence of causality. These studies instead underscore the need for nationally linked, individual-level datasets that allow rigorously assessing vaccine effectiveness and safety.

Beyond vaccination, a better understanding of COVID-19 immunopathology is essential. A systematic review included in this Research Topic summarizes the cumulative knowledge of cytokine dysregulation, endothelial injury, complement activation, and autoimmune mechanisms that contribute to disease severity and persistent immune dysfunction. These interconnected pathways help explain the clinical COVID-19 heterogeneity and provide potential targets for both therapeutic intervention and future vaccine development.

An important contribution to this Research Topic is its recognition that many questions arising in the post-pandemic period cannot be answered using conventional surveillance data alone. The papers examining persistent, excess mortality in Japan illustrate this challenge. Excess mortality is influenced by numerous interacting factors, including demographic changes, healthcare disruptions, delayed diagnoses, chronic disease burden, behavioral changes, and direct or indirect effects of the SARS-CoV-2 infection. Ecological studies can generate important hypotheses but cannot, solely, establish causal relationships. Progress will depend on the availability of comprehensive, linked datasets that integrate clinical information, vaccination history, healthcare resources, and mortality outcomes.

Thus, the contributions to this volume demonstrate that SARS-CoV-2 vaccination research has matured. The priority is no longer rapid development of vaccines but determining how vaccination strategies can be refined for different populations, adapted to incessant viral evolution, and evaluated using robust clinical and epidemiological evidence. Achieving these goals will require continual collaboration among immunologists, clinicians, epidemiologists, vaccinologists, and public health researchers.

Although COVID-19 is no longer considered a global public health emergency, it challenges the clinicians and public health experts continually. The studies presented in *SARS-CoV-2 Vaccines Beyond the Pandemic Era – Volume II* identify important knowledge gaps while showing further progress. Future research into vaccine design, immune protection, safety, and implementation will be essential for refining vaccination policies, improving long-term population protection, and strengthening preparedness against SARS-CoV-2 and future, potentially emerging infectious diseases. We hope that this Research Topic will stimulate further multidisciplinary studies and support the translation of evidence-based vaccination strategies to enhance the global preparedness for continuing challenges posed by SARS-CoV-2 or other emerging infectious diseases.

